# Regulation of the *Neurospora* Circadian Clock by the Spliceosome Component PRP5

**DOI:** 10.1534/g3.119.400500

**Published:** 2019-09-11

**Authors:** Huan Ma, Lin Zhang, Xinyang Yu, Yufeng Wan, Dongni Wang, Weirui Shi, Meiyan Huang, Manhao Xu, Enze Shen, Menghan Gao, Jinhu Guo

**Affiliations:** * State Key Laboratory of Biocontrol, Key Laboratory of Gene Engineering of the Ministry of Education, School of Life Sciences, Sun Yat-sen University, Guangzhou 510006, China,; † Zhuhai Interventional Medical Center, Zhuhai Precision Medical Center, Zhuhai People’s Hospital, Zhuhai Hospital Affiliated with Jinan University, Zhuhai, Guangdong 519000, China, and; ‡ School of Life Sciences, The Chinese University of Hongkong, Hongkong 999077, China

**Keywords:** circadian clock, *Neurospora crassa*, PRP5, spliceosome, alternative splicing

## Abstract

Increasing evidence has pointed to the connection between pre-mRNA splicing and the circadian clock; however, the underlying mechanisms of this connection remain largely elusive. In the filamentous fungus *Neurospora crassa*, the core circadian clock elements comprise White Collar 1 (WC-1), WC-2 and FREQUENCY (FRQ), which form a negative feedback loop to control the circadian rhythms of gene expression and physiological processes. Previously, we have shown that in *Neurospora*, the pre-mRNA splicing factors Pre-mRNA-processing ATP-dependent RNA helicase 5 (PRP5), protein arginine methyl transferase 5 (PRMT5) and snRNA gene *U4-2* are involved in the regulation of splicing of *frq* transcripts, which encode the negative component of the circadian clock system. In this work we further demonstrated that repression of spliceosomal component sRNA genes, *U5*, *U4-1*, and *prp5*, affected the circadian conidiation rhythms. In a *prp5* knockdown strain, the molecular rhythmicity was dampened. The expression of a set of snRNP genes including *prp5* was up-regulated in a mutant strain lacking the clock component *wc-2*, suggesting that the function of spliceosome might be under the circadian control. Among these snRNP genes, the levels of *prp5* RNA and PRP5 protein oscillated. The distribution of PRP5 in cytosol was rhythmic, suggesting a dynamic assembly of PRP5 in the spliceosome complex in a circadian fashion. Silencing of *prp5* caused changes in the transcription and splicing of NCU09649, a clock-controlled gene. Moreover, in the clock mutant *frq^9^*, the rhythmicity of *frq* I-6 splicing was abolished. These data shed new lights on the regulation of circadian clock by the pre-RNA splicing, and PRP5 may link the circadian clock and pre-RNA splicing events through mediating the assembly and function of the spliceosome complex.

Most organisms possess circadian clocks to orchestrate their daily metabolic, physiologic and behavioral rhythmicities (Bell-Pedersen *et al.* 1996). In eukaryotes, circadian clocks are controlled by positive and negative components that constitute transcriptional-translational negative feedback loops (Bell-Pedersen *et al.* 1996). In recent decades, regulatory layers acting on circadian clock networks, including post-transcriptional, post-translational and epigenetic regulation, have been identified. All of these regulations are necessary for the coordination of appropriate circadian clock functions (Bell-Pedersen *et al.* 1996; Gallego and Virshup 2007; Vanselow and Kramer 2007; Cibois *et al.* 2010; Sanchez *et al.* 2010; Durgan *et al.* 2011; Kojima *et al.* 2011; Staiger and Green. 2011; Staiger and Köster 2011; Kusakina and Dodd 2012; Wang *et al.* 2013).

The filamentous fungus *Neurospora crassa* is an important model for circadian research. In the *Neurospora* circadian clock, WC-1 and WC-2 are two PAS (PER-ARNT-SIM) domain-containing proteins that form the White Collar Complex (WCC). WCC binds to the promoter of the *frequency* (*frq*) gene and consequently activates its transcription. As a negative element, FRQ forms the FRQ-FRH complex (FFC) with FRH (FRQ-interacting RNA helicase) which inhibits the function of WCC. The inhibition of WCC by FRQ is relieved after temporal phosphorylation and degradation of FRQ. These positive and negative components constitute the transcription-translational negative feedback loop (TTFL) (Baker *et al.* 2012).

FRQ proteins contain small FRQ (s-FRQ) or large FRQ (l-FRQ) isoforms which are produced through the alternative splicing of intron 6 (I-6) of the *frq* pre-mRNA. The proportion of s-FRQ to l-FRQ is critical for the function of the clock (Liu *et al.* 1997). Higher temperatures induce more expression of l-FRQ and repress the expression of s-FRQ (Liu *et al.* 1997; Garceau *et al.* 1997; Colot *et al.* 2005; Diernfellner *et al.* 2005; Brunner and Diernfellner 2006). S-FRQ supports a longer circadian period and l-FRQ supports a shorter one (Liu *et al.* 1997; Brunner and Diernfellner 2006). FRQ isoforms also display differences in nucleocytoplasmic shuttling, in which l-FRQ accumulates in the nucleus (Cha *et al.* 2014). Recently, it has been shown that the catalytic subunit of exosome complex, RRP44, regulates the splicing of *frq* in addition in its mediating *frq* mRNA decay (Guo *et al.* 2009; Zhang *et al.* 2015). In *Neurospora*, the core nonsense-mediated RNA decay (NMD) factor - UPF1 - is also involved in controlling the splicing of *frq* I-6 (Wu *et al.* 2017).

A growing body of evidence suggests that alternative splicing plays a critical role in the regulation of circadian clocks in multiple species (Smith *et al.* 1989; Liu *et al.* 1997; Liu *et al*. 1998; Majercak *et al.* 2004; Colot *et al.* 2005; Diernfellner *et al.* 2005; Diernfellner *et al.* 2007; Hong *et al.* 2010; James *et al.* 2012a; James *et al.* 2012b; Jones *et al.* 2012; Koike *et al.* 2012; Low *et al.* 2012; McGlincy *et al.* 2012). The splicing regulation of clock genes or clock-controlled genes has been reported in various organisms, including mammals (Koike *et al.* 2012; Na *et al.* 2012), insects (Majercak *et al.* 2004; Sanchez *et al.* 2010) and plants (Sanchez *et al.* 2010; Hong *et al*. 2010; Jones *et al.* 2012; Wang *et al.* 2013; Schlaen *et al.* 2015). Most of the reported regulators are either spliceosome components or spliceosome-associated factors. The regulation of splicing acts as a critical layer on top of the basic negative feedback loop of the circadian clock.

Though connections between the circadian clock and pre-mRNA splicing have been investigated, the mechanism underlying the regulation of the spliceosome by the circadian clock remains largely unknown. PRP5 is a DExD/H-box containng RNA-dependent ATPase required for the formation of pre-spliceosome during the nuclear pre-mRNA splicing (Kosowski *et al.* 2009). PRP5 has been previously shown to mediate the splicing of *frq* I-6 (Zhang *et al.* 2015). We report that PRP5 is controlled by circadian clock, while in return also modulates the circadian oscillator and downstream alternative splicing events.

## Materials and Methods

### Strains and growth conditions

The *301-5* (*bd*, *a*) strain was used as the wild type (WT) strain. The *frq^9^* strain bears a frameshift mutation in the *frq* ORF (Aronson *et al.* 1994), and the *frq* gene is deleted in the *frq10* strain (Aronson *et al.* 1994). The *301-6-6* strain (*bd*, *his-3*, *A*) was used as the host strain for *his-3* targeting constructs. Liquid cultures were incubated in minimal medium (1 × Vogel’s, 2% glucose). When quinic acid (QA) was used, liquid cultures were grown in 0.01 M or indicated concentrations of QA (pH 5.8), 1 × Vogel’s, 0.1% glucose, and 0.17% arginine. The race tube medium contained 1 × Vogel’s, 0.1% glucose (0% when QA was used), 0.17% arginine, 50 ng/mL biotin, and 1.5% agar.

To generate these knockout (*KO*) strains, the entire coding sequences of snRNA genes *U5* and *U4-1* were deleted by replacement with the *hph* gene (Colot *et al.* 2006). The *Neurospora crassa* unit (NCU) numbers of *U5* and *U4-1* are NCU02572 and NCU09547, respectively. The gene replacement cassette harboring *hph* was transformed into the *bd*, *ku70^RIP^* strain.

The ds*prp5* strains were generated by introducing plasmids expressing RNA hairpins that were complementary to the gene to be inhibited into the WT strain *301-6-6* (Cheng *et al.* 2005), and this strain has been previously described (Zhang *et al.* 2015). The following primers containing specific restriction enzyme sites were used to generate the construct expressing RNA hairpins: forward: 5′-caggaattccgacgatgtgaggatgattcag-3′; reverse: 5′-aataagcttcgccgatatcgcgaccgggatc-3′. The hairpin sequence in the amplified products was complementary to approximately 500 bp of the gene of interest downstream of the *qa-2* promoter. The resulting plasmids were targeted to the *his-3* locus by transformation into *301-6-6* (*bd*, *his-3*, *A*). Addition of QA induces the repression of *prp5* expression in the ds*prp5* strain.

The information of other primers used in this work is available in the supplemental primer list.

### Luciferase assay for circadian rhythms

The *bar-frq-luc-I* plasmid was transformed into the *301-5* (WT) and ds*prp5* strains to monitor the real-time fluctuation of luciferase signal. Obtained transformants were screened using basta/ignite (200 μg/mL) resistance conferred by the *bar* gene (Gooch *et al.* 2008). To observe fluorescence the strains were inoculated on AFV (autoclaved FGS-Vogel’s) medium that contained 1×FGS (0.05% fructose, 0.05% glucose, 2% sorbose), 1 × Vogel’s medium, 50 μg/L biotin, and 1.8% agar. Firefly luciferin (BioSynt L-8200 D-luciferin firefly (synthetic) potassium salt) was added to the medium after autoclaving (final concentration of 50 μM). A LumiCycle high-throughput luminometer (Actimetrics, USA) was used for the luciferase assay as described previously (Gooch *et al.* 2008; Zhou *et al.* 2013).

### RNA and protein analyses

For reverse transcription PCR (qRT-PCR) analysis, the total RNA samples were isolated and treated with RNase-Free **DNase I (NEB, USA)** and subjected to reverse transcription using M-MLV **(Invitrogen, USA)** and random primers. The PCR products were resolved on a 1% agrose gel. The information of primers used in this work is available in the supplemental primer list.

For quantitative reverse transcription PCR (qRT-PCR) analysis, the total RNA samples were isolated and treated in same way described above. The obtained cDNAs were amplified with SYBR Green Master Mix (Takara, Japan) using a LightCycler 480 (Roche, Germany).

Protein extraction, western blot analysis and immunoprecipitation assays were performed as previously described (Garceau *et al.* 1997). Equal amounts of total protein (40 μg) were loaded in each lane of an SDS-PAGE gel (7.5%, containing a ratio of 37.5:1 acrylamide/bisacrylamide). Dephosphorylation of the FRQ protein was achieved by λ-phosphatase treatment.

### Chromatin Immunoprecipitation (ChIP) assay

The ChIP assay was performed as previously described (Cao *et al.* 2018). The immunoprecipitation was performed with a WC-2 antibody. Each experiment was independently performed three times, and immunoprecipitation without the WC-2 antibody or with the *wc-2^KO^* extract was used as the negative control. The following primers were used in ChIP assay: forward, 5′-tgtccaagcgggaagctggagt-3′; reverse, 5′-ccacgcttagggtaagtaactg -3′.

### Sucrose fractionation analysis

Sucrose density gradients (10–30%) were prepared and 4 mg of total protein samples were loaded for each analysis. The gradients were centrifuged at 175,000 × g for 18 h in a SW-40 rotor at 4°. Twelve equal fractions were collected and 450 μl of each fraction was used for RNA analysis. The samples were treated with DNase I prior to the RT-PCR to determine the levels of *U5*. Western blot analysis was also used to determine the distribution of PRP5 (Wu *et al.* 2017).

### Statistical analysis

Statistical significance was calculated using Student’s *t*-test. The values presented are the mean ± SD or SE as denoted. Significance values are *P < 0.05, **P < 0.01 and ^#^*P* < 0.001.

### Data availability

The RNA sequencing data of *Neurospora* WT strain in constant darkness for 12 hr (DD12) and 20 hr (DD20) were deposited at Gene Expression Omnibus (GSE117118). Supplemental protocol for RNA-seq analysis and Tables S1-S5 are available at FigShare: https://doi.org/10.25387/g3.9790751.

## Results

### Regulation of the circadian clock by PRP5

**Figure 1:**
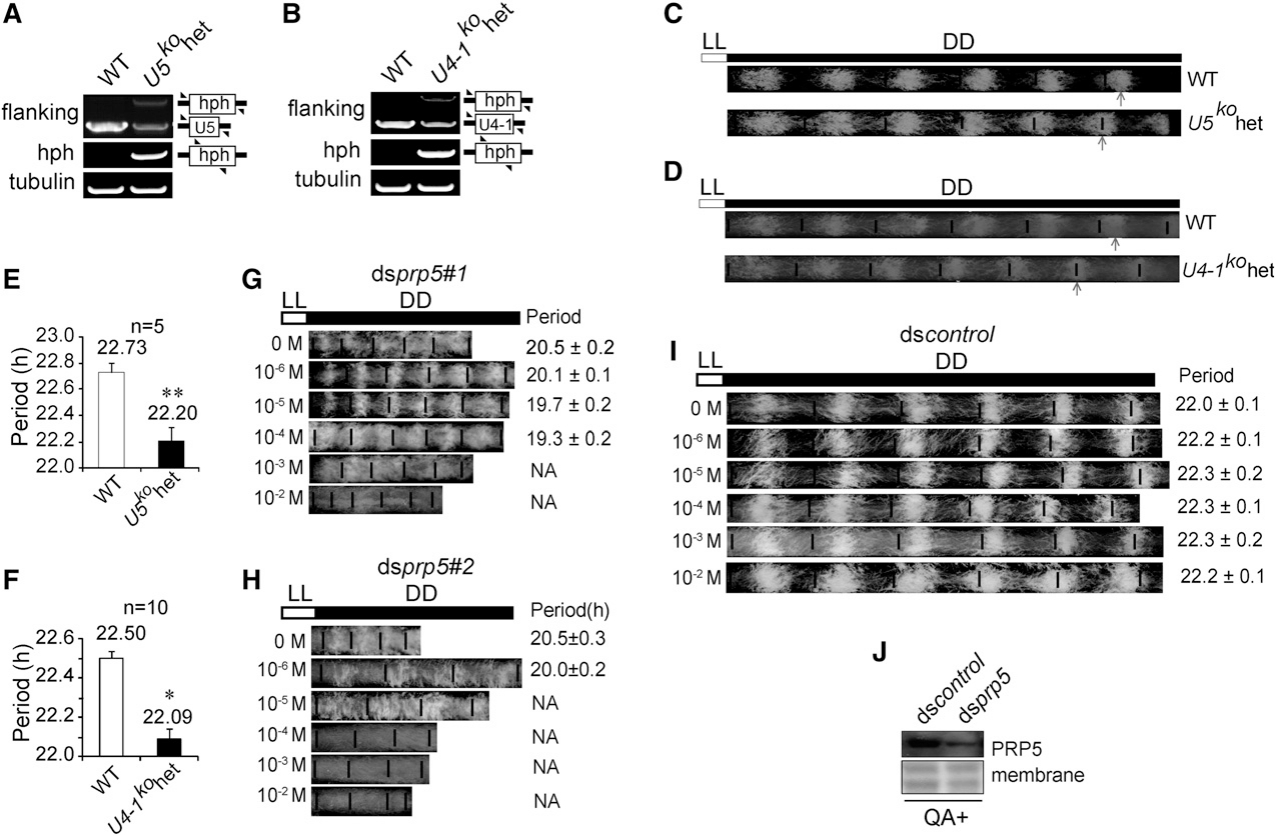
Spliceosome components regulate the circadian clock. (A&B) PCR validation of the *U5* and *U4-1* genes in the *U5^KO^* (A) and *U4-1^KO^* (B) strains, respectively. The PCR results showed the presence of *hph* in both knockout strains but not in WT, and decrease in the target gene abundance, which suggest that these two strains are heterokaryons. Arrows denote the locations of relative PCR primers (C&D) Race tube assay of *U5^KO^* heterokaryon (C) and *U4-1^KO^* (D) heterokaryon strains under constant dark. Arrows indicate the location of the six conidiation bands of each strain. (E&F) Statistics of the circadian periods. The values are presented as the mean ± SD, n = 3. (G-I) Race tube assay results of two transformants of ds*prp5* strain (G&H) and ds*control* (I) at different concentrations of QA. (J) Western blot validation of the ds*prp5* strain. Membrane stained with amido black served as control.

Spliceosome is one of the largest cellular complexes and comprises small nuclear ribonucleic acids (snRNAs), small nuclear ribonucleoprotein particles (snRNPs) and an additional group of non-snRNP proteins. *Neurospora* possesses 15 snRNA genes (belonging to the *U1*, *U2*, *U5*, and *U4/U6* species) in total (Wan *et al.* 2015). To determine the effects of the spliceosomal components on the circadian clock, knockout strains of the snRNA genes *U5* (*U5^KO^*) and *U4-1* (*U4-1^KO^*) were created and validated (Figure 1A&B). These two knockout strains are heterokaryon that failed to cross and generate homokaryotic progeny, suggesting that appropriate pre-mRNA splicing is essential for sexual reproduction. To analyze the effects of the *U4-1^KO^* and *U5^KO^* heterokaryotic strains on the circadian rhythms, these two strains were inoculated inside and at one end of long glass tubes called race tubes. In a race tube assay, *Neurospora* grows toward the other end of the tube on a layer of solid media. During growth, *Neurospora* releases asexual conidia, and the circadian periods can be calculated by analyzing the interval time between the conidiation bands (Baker *et al.* 2012). From the race tube results, both *U4-1^KO^* and *U5^KO^* heterokaryotic strains exhibited a slight but significant decrease in their conidiation period lengths (Figure 1C-F).

Previously we generated a knockdown strain of the *prp5* gene, which has been named ds*prp5* (Zhang *et al.* 2015). We have obtained two transformants of ds*prp5*, which are named ds*prp5#*1 and ds*prp5#2*, and the ds*prp5#1* strain has been previously described (Zhang *et al.* 2015). In these two transformants, QA induces the silencing of *prp5* expression. Both of the ds*prp5* strains display much slower growth rate compared to WT even without QA, and the presence of 0.01M QA resulted in a more dramatic decrease in growth and a reduction of aerial hyphae and conidia, which might be owing to a leakage effect of the *qa* promoter leakage. Despite the conferred growth, ds*prp5* exhibited conidiation rhythms in the absence of QA, with a period shorter compared to that in WT strain. By contrast, the conidiation rhythms of ds*prp5* were abolished in the presence of QA (Figure 1G-I), suggesting that knockdown of *prp5* leads to influence on the circadian clock. We used ds*prp5#1* for the following studies as it shows slighter leakage effect. Previously the repression of *prp5* RNA in this strain was verified by northern blot, and here, we further validated them by western blot with PRP5 antiserum (Figure 1J).

**Figure 2:**
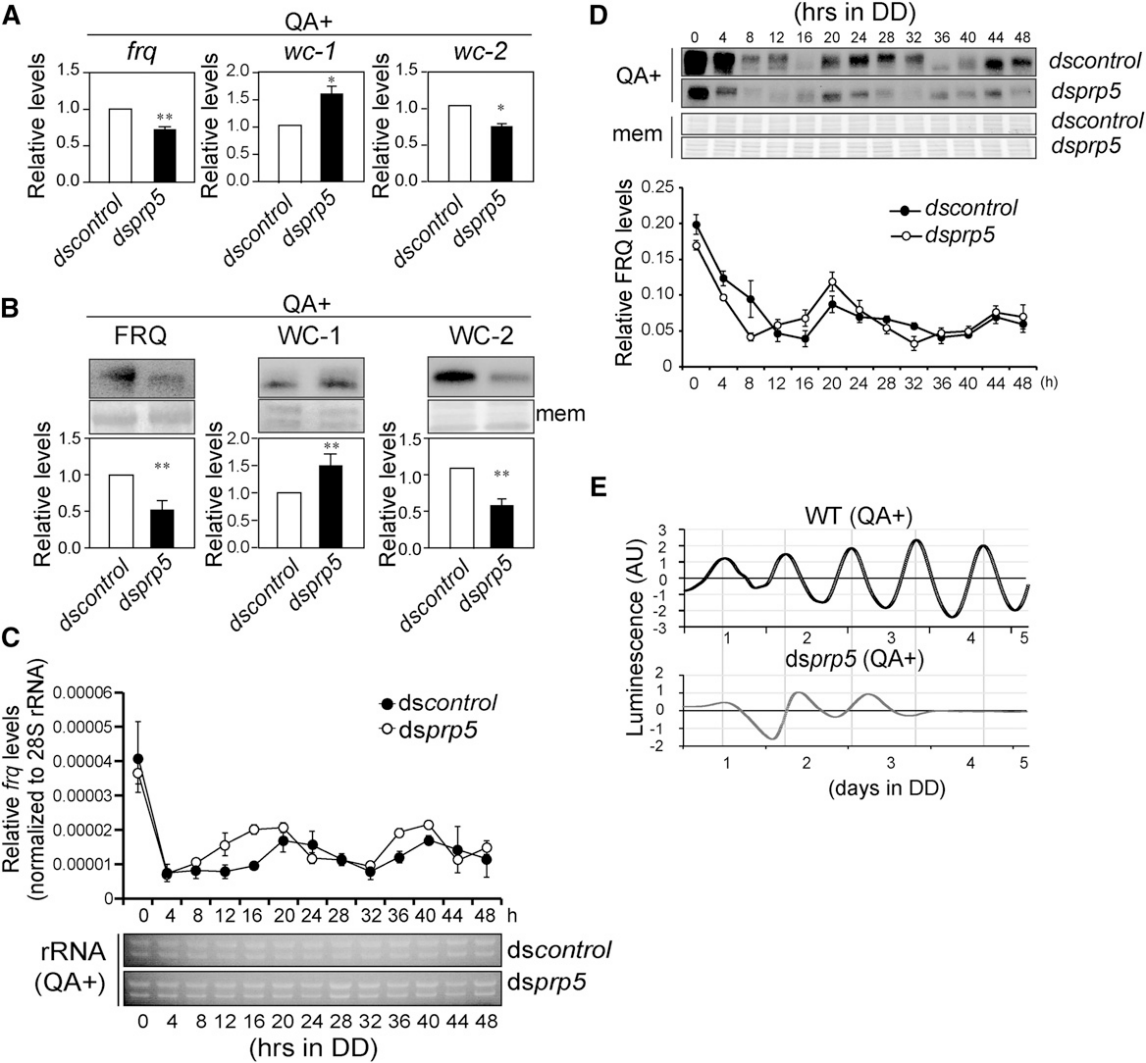
PRP5 regulates *Neurospora* circadian rhythms. (A) qRT-PCR results of *frq*, *wc-1* and *wc-2* in ds*control* and ds*prp5* strains. The strains were grown in constant light (LL). The expression was normalized to 28s rRNA. Values are mean ± SD, n = 5. (B) Western blot results of FRQ, WC-1 and WC-2 in ds*control* and ds*prp5* strains. The strains were grown in LL. Values are mean ± SD, n = 5. (C) qRT-PCR analysis showing the expression of *frq* RNA in ds*prp5* in constant dark over a 48-h time course. Electrophoresis results of RNA samples were shown as control. The expression was normalized to 28s rRNA. The values are presented as the mean ± SD, n = 3. (D) Western blot analysis of the FRQ protein levels in ds*prp5* in constant darkness over a 48-h time course. The values are presented as the mean ± SD, n = 3. (E) Representative results of luciferase reporter assays showing the *frq* promoter activity of the indicated strains in constant darkness. The measurement of luciferase activity was normalized by subtracting the baseline luciferase signal.

In ds*prp5* strain, alterations in the expression of the clock genes *frq*, *wc-1* and *wc-2* in constant light were observed. The RNA levels of *frq* and *wc-2* were decreased while *wc-1* increased in ds*prp5*. The changes in protein levels of these three genes were consistent with the RNA data (Figure 2A&B). These data suggest that the negative feedback loop of the circadian clock might be extensively affected upon *prp5* knockdown. We next examined the expression of the *frq* mRNAs and FRQ proteins in constant dark (DD) for 48 hr, by qRT-PCR and western blot analysis, respectively. The qRT-PCR results showed that the *frq* mRNA levels oscillated and the period was ∼2 h shorter in ds*prp5* than that in ds*control* (Figure 2C). The western blot analysis revealed that FRQ proteins showed a peak at DD20 but the second peak was dampened (Figure 2D). The results of both *frq* RNA and FRQ protein showed that the phase of rising up on the first day after transition from LL to DD was advanced in the ds*prp5* strain (Figure 2C, D). We next introduced a luciferase reporter construct under the control of the *frq* promoter into the WT and ds*prp5* strains, to allow us to observe the molecular rhythms for a longer time, and the results showed that the rhythmicity of luciferase activity was severely dampened in the ds*prp5* which disappeared within several days (Figure 2E). These results demonstrate that the spliceosome plays an important role in maintaining the robust circadian rhythms.

### Circadian control of PRP5 gene expression

**Figure 3:**
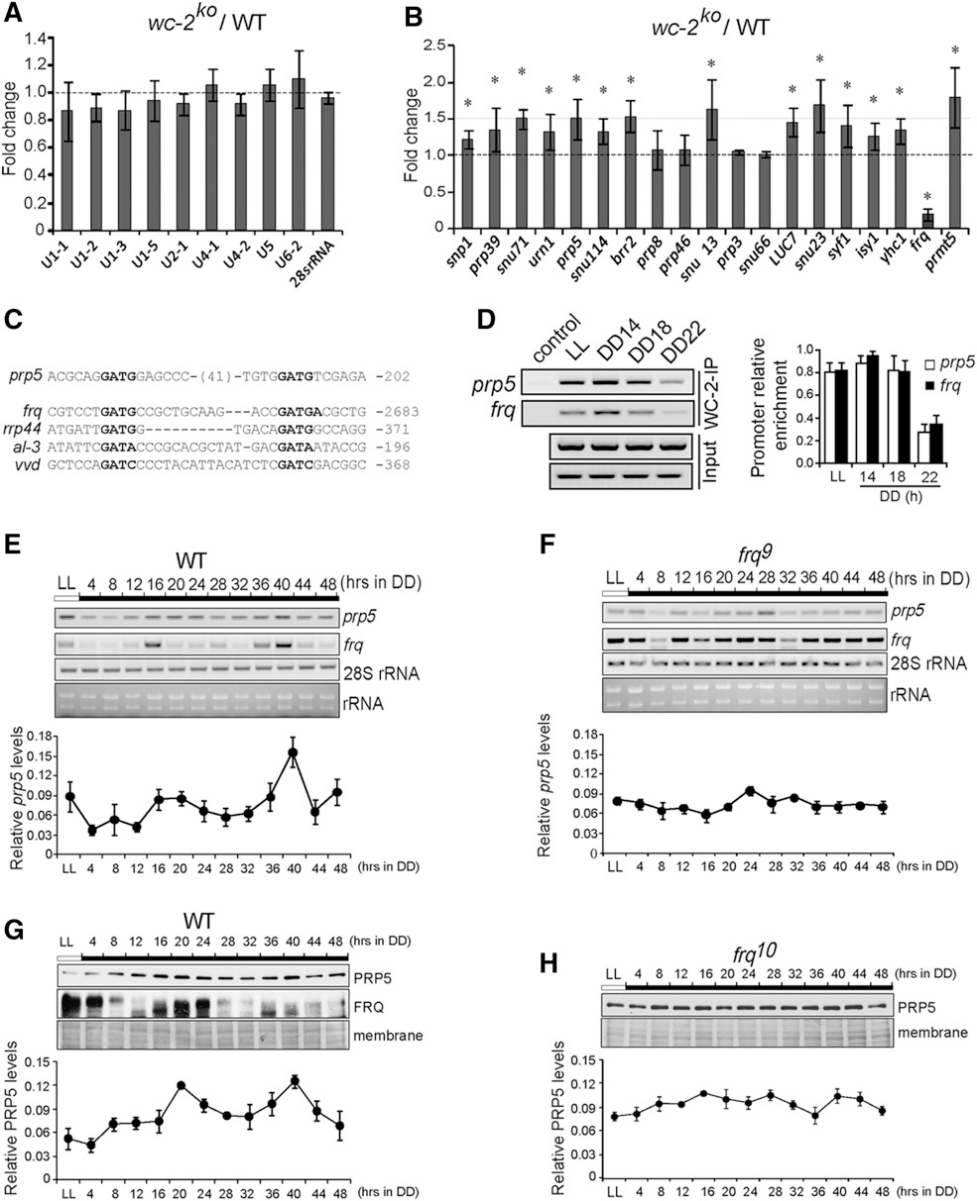
Circadian clock controls expression of spliceosomal genes. (A) qRT-PCR of snRNA genes in the *wc-2^KO^* strain. The expression was normalized to 28s rRNA. The values are presented as the mean ± SD, n = 3. (B) qRT-PCR of snRNP genes in the *wc-2^KO^* strain grown in LL. The values were normalized to the levels in the WT strain. The values are presented as the mean ± SD, n = 3. (C&D) ChIP assays with WC-2 antibody showing that WCC binds to the *prp5* and *frq* promoters specifically and rhythmically in the wild-type strain. The values are presented as the mean ± SD, n = 3. (E&F) qRT-PCR results showing the expression of *prp5* in WT and *frq9* strains. The expression was normalized to 28s rRNA. The values are presented as the mean ± SD, n = 3. (G&H) Western blot analysis of the PRP5 protein levels in the WT (G) and *frq^10^* (H) strain in constant darkness over a 48-h time course. The values are presented as the mean ± SD, n = 3.

To assess whether the expression of spliceosomal genes is affected by the circadian clock, we compared the expression of spliceosomal genes in the *wc-2* knockout strain (*wc-2^KO^*) using qRT-PCR. The expression of all of the tested snRNA genes showed no significant changes, while the expression of most snRNP genes was increased, with the exception of that of *prp8*, *prp46*, *prp3* and *snu66* (Figure 3A, B). Despite the relatively low levels, the overall increase suggests that the gene expression and function of spliceosome components may be under the circadian control.

Sequence analysis indicated the existence of a putative C-box in the promoter region of *prp5* (Figure 3C). Flanking primers were synthesized, and a chromatin immunoprecipitation (ChIP) assay was conducted, and the results showed that WCC bound specifically to the C-box-like element in the *prp5* promoter and this binding might peak around DD14 (Figure 3C, D). These data suggest that the expression of PRP5 might be controlled by the circadian clock.

We next investigated *prp5* expression under constant dark conditions for 48 h. The results of the RT-PCR analyses showed that *prp5* RNA levels exhibited low but significant circadian rhythmicity in the WT strain. By contrast, the circadian rhythmicity of *prp5* RNA was abolished in the *frq^9^* strain (Figure 3E, F). In consistence, the western blot results using a PRP5 antibody demonstrated that the PRP5 levels oscillated with a period of approximately 24 h under constant dark in the WT strain but not in the *frq^10^* strain, in which the ORF region of *frq* gene was deleted (Figure 3G, H). These data confirmed the clock-controlled expression of *prp5* RNA and PRP5 protein.

### The circadian clock controls PRP5 assembly

**Figure 4:**
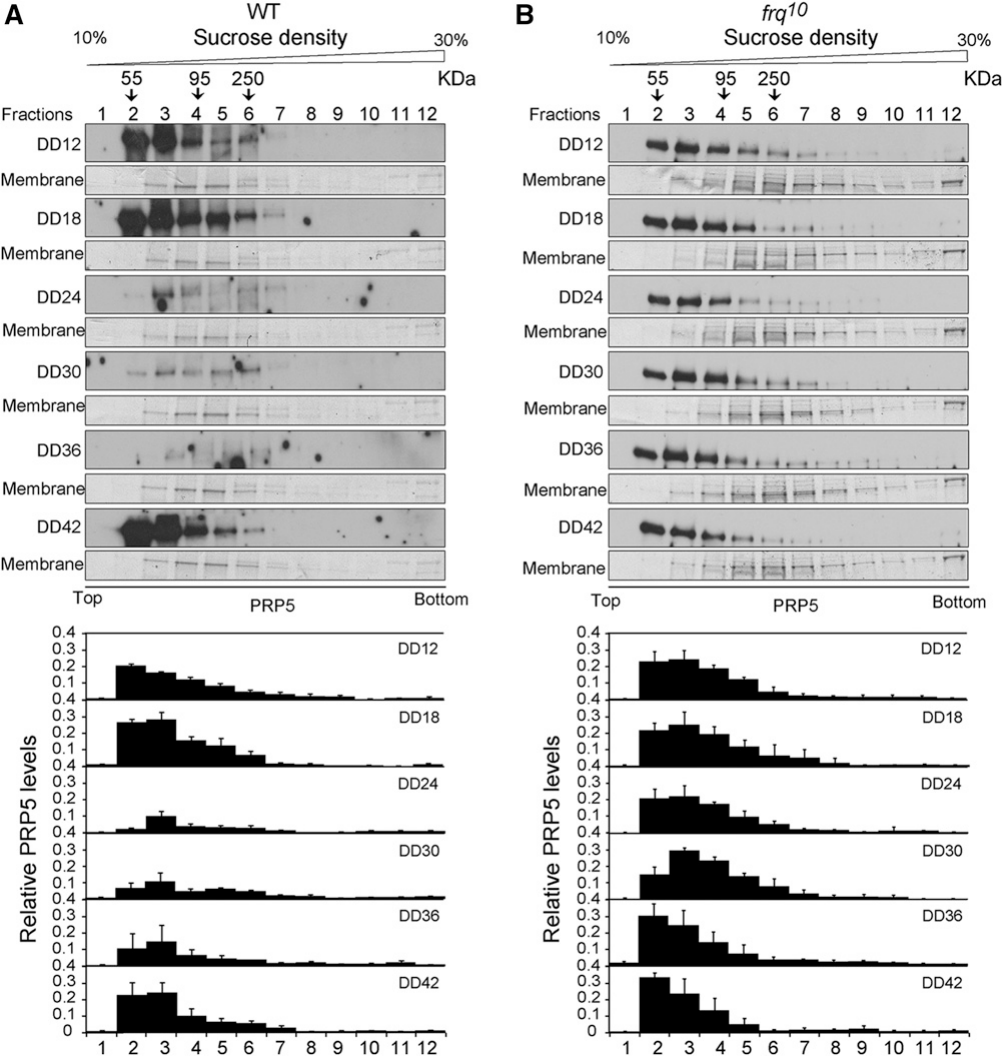
Spliceosomal assembly is under circadian control. (A&B) The distribution of PRP5 in sucrose fractionation samples from DD12 to DD42 in increments of 6 h, in WT (A) and *frq^10^* (B) strains. Upper: Representative western blot results of PRP5 in sucrose fractionated samples at each time points are shown. Bottom: Densitometric quantification of the PRP5 distribution in fractions at different time points. For comparison, all 72 samples were blotted onto one membrane after electrophoresis. The total value from all 12 densitometric traces of each experiment was normalized to be 1.0. The values are presented as the mean ± SD, n = 3.

We next conducted sucrose fractionation assays to examine whether the circadian clock controls the assembly of PRP5 in the spliceosome complex, which can be reflected by the changes in PRP5 distribution in fractionated samples (Wu *et al.* 2017). We performed sucrose sedimentation assays in triplicate and compared the distribution of PRP5 in the sucrose gradient fractions as a function of time under constant dark conditions. A periodicity of approximately 24 h was observed in the WT strain while it was arrhythmic in *frq10* (Figure 4A, B). These data suggest that circadian clock governs the assembly of PRP5 in the spliceosome complex.

### Regulation of the splicing rhythm by the spliceosome and clock

**Figure 5:**
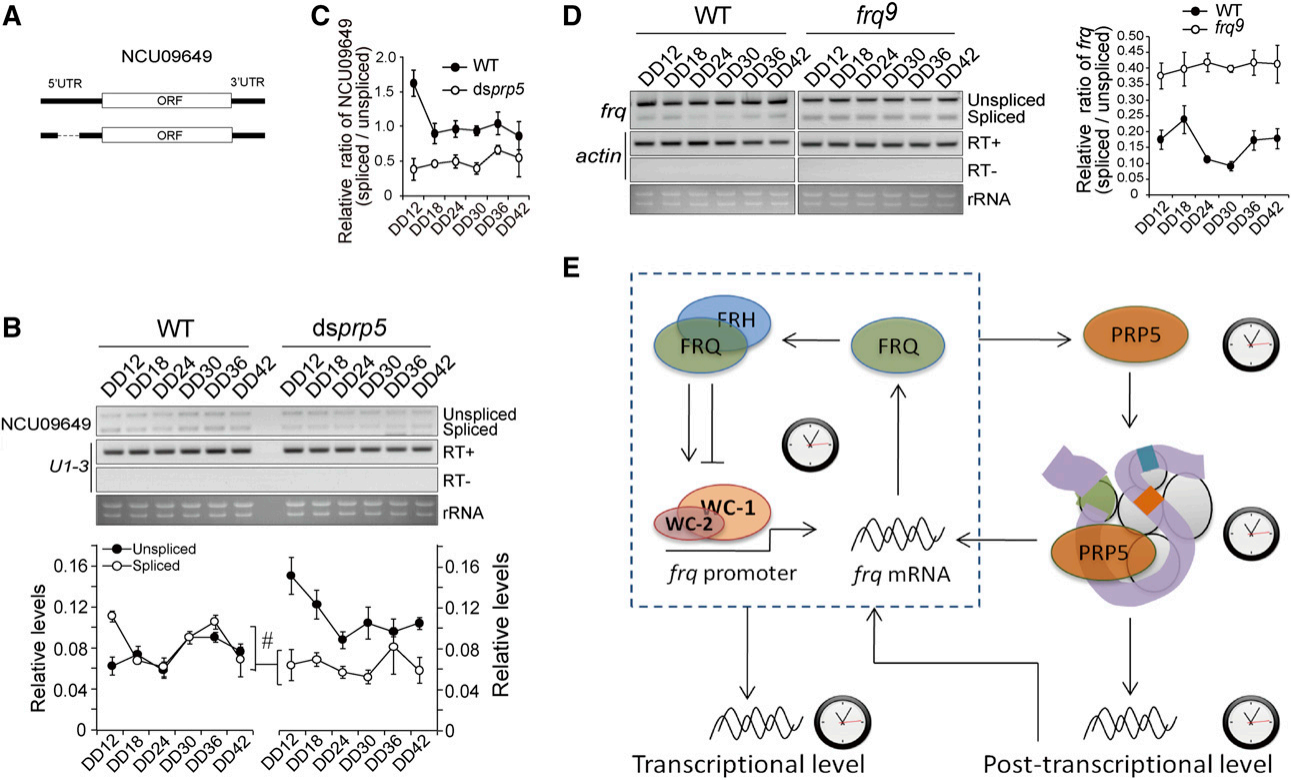
PRP5 controls the circadian rhythm of splicing. (A) Schematic representation of NCU09649 splicing isoforms. (B) RT-PCR analysis of the alternative splicing of NCU09649 at DD12 to DD42 in increments of 6 h in the WT and *dsprp5* strains. The values are presented as the mean ± SD, n = 3. (C) Densitometric quantification of the results. The values are presented as the mean ± SD, n = 3. (D) RT-PCR analysis of the alternative splicing of *frq* I-6 at DD12 to DD42 in increments of 6 h in WT and *frq^9^* strains. The values are presented as the mean ± SD, n = 3. (E) The oscillator of the *Neurospora* circadian clock consists of positive and negative elements including FRQ, FRH, WC-1 and WC-2, which constitute the transcriptional-translational negative feedback loop. The protein levels and assembly of PRP5 are controlled by the circadian clock. PRP5 regulates circadian in at least two pathways: 1) feeds back to regulate the splicing of *frq* and 2) mediates the rhythmic splicing events of a set of downstream genes.

To identify the downstream genes whose splice variant proportions are controlled by the circadian clock, RNA-sequencing (RNA-seq) and bioinformatic analyses were carried out. The duplicate RNA samples from WT grown in constant darkness for 12 hr (DD12) and 20 hr (DD20) were used to generate the mRNA-seq library and RNA, each of which comprised equally pooled three independent samples (GSE117118). From this analysis, we identified hundreds of sites that were differentially spliced when DD12 and DD20 were compared (Supplemental Protocol and Tables S1-S5). We further conducted qRT-PCR in about thirty splicing sites in three set of samples harvested at DD12 to DD42 in increments of 6 h, however, only a few of which were confirmed to oscillate. This inconsistency suggests the differences between two DD12 and DD20 mostly represent non-circadian fluctuations. Among these genes, NCU09649 encodes a putative metallophosphoesterase that contains only one intron in its 5′ UTR region (Figure 5A). We investigated the splicing of NCU09649 in the WT and ds*prp5* strains under constant dark at DD12 through DD42. The RT-PCR results showed that both the unspliced and spliced species of NCU09649 oscillated in WT but not in ds*prp5* (Figure 5B&C). Compared to the WT, the rhythmicity of the spliced transcripts of NCU09649 was significantly dampened in ds*prp5*. In addition, in the ds*prp5* strain, the levels of spliced species were significantly decreased (Figure 5B, C), suggesting a role of PRP5 in the regulation of NCU09649 splicing.

In the *Neurospora* circadian clock, seven alternative splice variants of the core clock gene *frq* are observed (Diernfellner *et al.* 2005). At the protein level, these variants yield two FRQ isoforms, s-FRQ and l-FRQ, depending on exclusion or inclusion of *frq* I-6, respectively. The *frq* open reading frame (ORF) has three putative initiation codons (AUG), of which only the first and third function in the initiation of translation (Liu *et al.* 1998; Colot *et al.* 2005). The protein product that is translated from the first initiation codon is l-FRQ, whereas s-FRQ is translated from the third initiation codon and lacks 99 amino acid residues from its N-terminus. Splicing of *frq* I-6 removes the first initiation codon so that the *frq* mRNA lacking I-6 is translated into s-FRQ (Liu *et al.* 1998; Colot *et al.* 2005; Diernfellner *et al.* 2005; Diernfellner *et al.* 2007; Neiss *et al.* 2008). We have previously reported that knockdown of *prp5* represses the splicing of *frq* I-6, suggesting that alternative splicing in part explains the dysregulation of the circadian clock in the ds*prp5* strain (Zhang *et al.* 2015).

Diernfellner *et al.* showed that splicing of *frq* I-6 displayed a rhythm under DD, suggesting that splicing of *frq* I-6 is under circadian control (Diernfellner *et al.* 2007). Here we further measured the levels of the spliced transcript variants in the WT and *frq^9^* strain, which bears a frame-shift mutation in the *frq* ORF and produces a truncated protein product with no circadian function (Aronson *et al.* 1994). RT-PCR using primers flanking *frq* I-6 was carried out to examine the expression of *frq* with spliced or unspliced I-6 under DD, in the WT and *frq9* strains (Figure 5D). Considering that the oscillation at the transcriptional level might mask the analysis of splicing rhythmicity, we calculated the ratio of spliced isoforms *vs.* unspliced isoform. The results showed that the ratio of spliced/unspliced transcripts oscillated in WT which is consistent with the previous observation (Diernfellner *et al.* 2007). In contrast, both the unspliced and spliced species showed no overt rhythms in *frq^9^* (Figure 5D).

## Discussion

In eukaryotes, the regulation of alternative splicing plays a critical role in regulating the normal rhythms of the circadian clock. The rhythmicity of spliced variants can be attributed to rhythmic transcription, rhythmic splicing or a combination of both (Koike *et al.* 2012; Partch *et al.* 2014; Lipton *et al.* 2015).

PRP5 is an RNA-dependent ATPase present in the commitment complex, which regulates pre-spliceosome formation and the release of spliced mRNA from the spliceosome. During splicing, Prp5p recruits U2 snRNP to pre-mRNA and hydrolyses ATP to stabilize the association of U2 in the pre-spliceosome in *Saccharomyces cerevisiae* (Kosowski *et al.* 2009). In this work, we revealed that PRP5 regulates the circadian rhythms of *Neurospora* may play a role in linking the circadian clock and downstream splicing events. Furthermore, some other spliceosomal factors, such as snRNA *U4-1* and *U5*, have also been implicated in the regulation of circadian clock. Repression of PRP5 and other spliceosome components resulted in differential influences on *frq* pre-mRNA splicing. For instance, knockdown of *prp5* and *U4-2* results in decreased *frq* I-6 splicing while knockdown of *prmt5* results in an increase in *frq* I-6 splicing (Zhang *et al.* 2015). For NCU09649, knockdown of *prp5* also led to decreased levels of spliced transcripts (Figure 3B), suggesting that PRP5 and *U4-2* act to promote spicing, while PRMT5 represses splicing.

Both the expression patterns of *prp5* RNA and PRP5 protein exhibited circadian rhythmicity (Figure 3E-H), moreover, we showed that the assembly of PRP5 in the spliceosome complex was governed by circadian clock (Figure 4). Taken together with its important role in pre-spliceosome formation (Kosowski *et al.* 2009), these findings suggest that circadian clock may regulate the composition and function of spliceosome and a set of splicing events as consequence.

FRQ is the core circadian regulator in *Neurospora*, and alternative splicing of *frq* I-6 is critical for the production of FRQ isoforms (Liu *et al.* 1997). The alternative splicing of *frq* I-6 can be affected by the ambient temperature and associated genes (Liu *et al.* 1997; Garceau *et al.* 1997; Colot *et al.* 2005; Diernfellner *et al.* 2005; Brunner and Diernfellner. 2006; Zhang *et al.* 2015). The alternative splicing of *frq* I-6 exhibited overt circadian rhythms (Figure 5D), in agreement with the previous findings (Diernfellner *et al.* 2007; Zhang *et al.* 2015), these data confirme that the splicing of *frq* I-6 is rhythmically governed by the circadian clock.

As for NCU09649, the levels of both unspliced and spliced transcripts oscillated (Figure 5B, C), but the ratio of the spliced *vs.* unspliced species showed no overt rhythmicity, suggesting that both the transcription and splicing of NCU09649 are under control of the circadian clock. Repression of *prp5* led to altered splicing patterns, suggesting that PRP5 plays an important role in mediating the pre-RNA splicing of NCU09649. Together, these findings suggest that PRP5 may bridge the circadian clock and alternative splicing through regulating the spliceosome function (Figure 5E).

The circadian period of the strain exclusively expressing l-FRQ is shorter compared to the WT strain which expresses l-FRQ and s-FRQ simultaneously while the strain exclusively expressing s-FRQ possesses a longer period (Liu *et al.* 1997). In this work, the heterokaryon knockout strains of *U4-1* and *U5* showed shorter circadian periods, which might be due to less splicing of *frq* I-6. Consistently, in absence of QA, ds*prp5* displayed a shorter period (Figure 1G,H). The ratio of *frq* transcripts containing I-6 is significantly increased in ds*prp5* strain (Zhang *et al.* 2015), however, the molecular rhythms ds*prp5* was too dampened to calculate the period (Figure 2E). These data suggest that in addition to *frq* I-6 splicing, other unknown regulators, which are likely potential PRP5 targets, might be involved in determining the abnormal periodicity (Figure 5E). Decoupling between different regulatory layers might occur in the control of circadian clock, for instance, the *fwd-1* null strain showed robust rhythms at the transcriptional level of *frq* but not the FRQ protein level (Larrondo *et al.* 2015). Though the conidiation rhythms and clock gene expression were affected in ds*prp5* strain, at the molecular level, it still showed rhythmicities revealed by *frq*/FRQ expression and luciferase reporter assay (Figure 1 and Figure 2), suggesting that decoupling might occur between the flow from circadian oscillator to the output.
